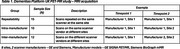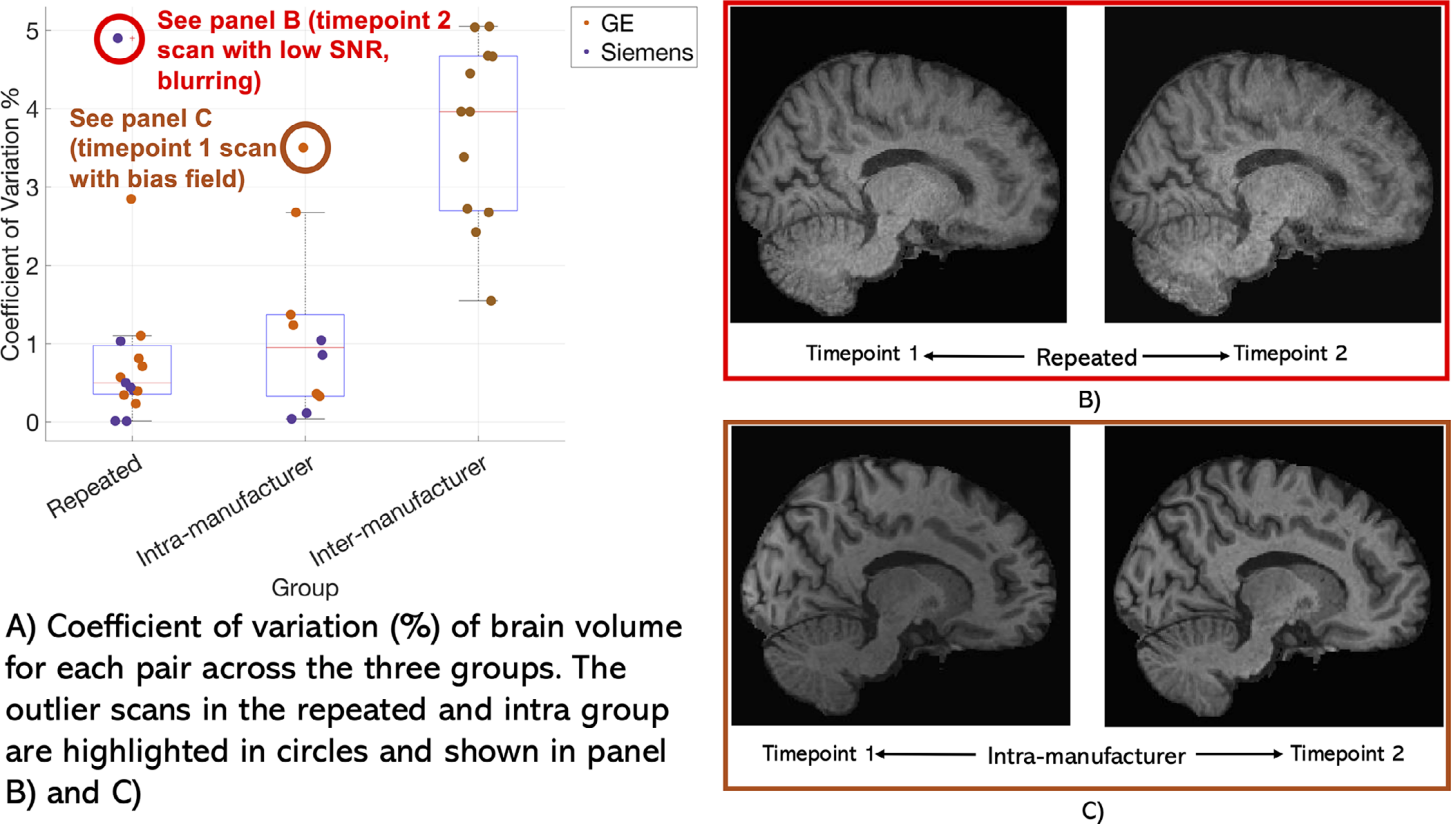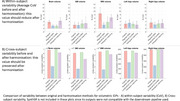# Harmonising Multisite Brain MRI Data: Insights from the Dementias Platform UK (DPUK) PET‐MR harmonisation Study

**DOI:** 10.1002/alz70856_097537

**Published:** 2025-12-24

**Authors:** Gaurav V Bhalerao, Pawel J Markiewicz, David L Thomas, Enrico de Vita, Laura Parkes, Gerard Thompson, Jane MacKewn, Georgios Krokos, Catriona Wimberley, William Hallett, Li Su, Stephen Smith, Paresh Malhotra, Nigel Hoggard, John‐Paul Taylor, Craig Ritchie, Joanna M Wardlaw, Paul M Matthews, Franklin I Aigbirho, John T O'Brien, Alexander Hammers, Nick C Fox, Karl Herholz, Frederik Barkhof, Karla Miller, Julian Matthews, Ludovica Griffanti

**Affiliations:** ^1^ Department of Psychiatry, Oxford Centre for Human Brain Activity (OHBA), Wellcome Centre for Integrative Neuroimaging, University of Oxford, Oxford, Oxfordshire, United Kingdom; ^2^ University College London, London, United Kingdom; ^3^ University College, London, United Kingdom; ^4^ King's College, London, London, United Kingdom; ^5^ University of Manchester, Manchester, United Kingdom; ^6^ University of Edinburgh, Edinburgh, United Kingdom; ^7^ Perceptive, London, London, United Kingdom; ^8^ University of Sheffield, Sheffield, United Kingdom; ^9^ University of Oxford, Oxford, United Kingdom; ^10^ Imperial College London, London, United Kingdom; ^11^ Newcastle University, Newcastle upon Tyne, United Kingdom; ^12^ University of Cambridge, Cambridge, United Kingdom; ^13^ King's College London, London, United Kingdom

## Abstract

**Background:**

The DPUK PET‐MR Harmonisation Study aims to quantify within‐site and across‐site variability of brain scans across scanning equipment. Brain images were acquired from 8 PET‐MR scanners to optimise clinical trial design and evaluate PET‐MR methods. Scans were collected from healthy elderly participants in three groups: Repeated, Intra‐manufacturer, and Inter‐manufacturer (Table 1). This study examines variability in imaging‐derived phenotypes (IDPs) from T1‐weighted MRI scans and evaluates harmonisation methods.

**Method:**

All T1w scans were processed using the UK Biobank (UKB) pipeline to extract volumetric IDPs (total brain, tissue‐specific, hippocampus). Within‐subject (scanner) variability was quantified using the Coefficient of Variation (CoV) for each subject pair and IDP.

In the inter‐manufacturer group, CoV and cross‐subject variability (biological variability measured as across subjects’ standard deviation of within‐subject mean IDP) were assessed following three harmonisation approaches:

IQM‐based: Identify image quality metrics (IQMs) (e.g., noise, contrast) that significantly differ across scanners and exhibit low correlation (‐0.5<r<0.5) with IDPs and regress these IQMs from IDPs.

IDP‐based: Scanner‐related batch effects were adjusted using longitudinal ComBat method (Beer et al. 2020).

Image‐based: Two techniques—(1) histogram matching (2) SynthSR (Iglesias et al. 2023)—were applied to harmonise T1w scans before UKB pipeline processing.

**Result:**

Visual inspection of outliers showed that quality of the scans highly impacts the within‐subject variability (Figure 1). Post‐harmonisation (Figure 2), IQM regression reduced within‐subject CoV (e.g., brain volume: 3.7±1.2% to 1.5±0.9%) but also showed reduced cross‐subject variability indicating loss of signal of interest. Longitudinal ComBat reduced CoV (e.g., brain volume: 3.7±1.2% to 1.4±0.8%) and preserved cross‐subject variability consistently across IDPs. Image‐based methods were inconsistent; histogram matching worked for some IDPs, while SynthSR outputs were not compatible with the downstream pipeline used e.g., failed brain extraction upon visual inspection.

**Conclusion:**

ComBat reduces IDP variability but requires specifying batch/site variables, limiting its use for other groups (e.g., intra‐manufacturer subjects are from different sites). IQM regression offers more flexibility without needing scanner details but needs refinement to address cross‐subject variability. Image‐based methods require further developments. Our study emphasises the importance of quantifying different aspects of variability when assessing harmonisation in multisite MRI studies, to improve consistent imaging biomarkers for early dementia diagnosis.